# Patterns and predictors of fear of childbirth and depressive symptoms over time in a cohort of women in the Pwani region, Tanzania

**DOI:** 10.1371/journal.pone.0277004

**Published:** 2022-11-03

**Authors:** Agnes Fredrick Massae, Margareta Larsson, Andrea Barnabas Pembe, Columba Mbekenga, Agneta Skoog Svanberg

**Affiliations:** 1 Department of Women’s and Children’s Health, Uppsala University, Uppsala, Sweden; 2 Department of Community Health Nursing, Muhimbili University of Health and Allied Sciences, Dar es Salaam, Tanzania; 3 Department of Obstetrics and Gynaecology, Muhimbili University of Health and Allied Sciences, Dar es Salaam, Tanzania; 4 School of Nursing, Hubert Kairuki Memorial University, Dar es Salaam, Tanzania; Sabzevar University of Medical Sciences, ISLAMIC REPUBLIC OF IRAN

## Abstract

**Background:**

Fear of childbirth (FoB) and depressive symptoms (DS) are experienced by many women and can negatively affect women during and after pregnancy. This study assessed patterns of FoB and DS over time and associations of postpartum FoB and DS with sociodemographic and obstetric characteristics.

**Methods:**

We conducted a longitudinal study at six health facilities in Tanzania in 2018–2019. Pregnant women were consecutively assessed for FoB and DS before and after childbirth using the Wijma Delivery Expectancy/Experience Questionnaire versions A & B and the Edinburgh antenatal and postnatal depressive scale. This paper is based on 625 women who completed participation.

**Results:**

The prevalence rates of FoB and DS during pregnancy were 16% and 18.2%, respectively, and after childbirth, 13.9% and 8.5%. Some had FoB (6.4%) and DS (4.3%) at both timepoints. FoB was strongly associated with DS at both timepoints (p < 0.001). Both FoB (p = 0.246) and DS (p < 0.001) decreased after childbirth. Never having experienced obstetric complications decreased the odds of postpartum and persisting FoB (adjusted odds ratio (aOR) 0.44, 95% confidence interval (CI) 0.23–0.83). Giving birth by caesarean section (aOR 2.01, 95% CI 1.11–3.65) and having more than 12 hours pass between admission and childbirth increased the odds of postpartum FoB (aOR 2.07, 95% CI 1.03–4.16). Postpartum DS was more common in women with an ill child/stillbirth/early neonatal death (aOR 4.78, 95% CI 2.29–9.95). Persisting DS was more common in single (aOR 2.59, 95% CI 1.02–6.59) and women without social support from parents (aOR 0.28, 95% 0.11–0.69).

**Conclusions:**

FoB and DS coexist and decrease over time. Identifying predictors of both conditions will aid in recognising women at risk and planning for prevention and treatment. Screening for FoB and DS before and after childbirth and offering psychological support should be considered part of routine antenatal and postnatal care. Furthermore, supporting women with previous obstetric complications is crucial. Using interviews instead of a self-administered approach might have contributed to social desirability. Also, excluding women with previous caesarean sections could underestimate FoB and DS prevalence rates.

## Introduction

Childbirth is an experience with many dimensions and is unique to each woman. It is normal to experience some apprehension during pregnancy and after childbirth. If the feelings affect women performing their daily activities, they might suffer perinatal mental health problems such as depression and fear of childbirth (FoB) [1]. Depressive symptoms (DS) and FoB are universal concerns that can influence one another and may occur before and/or after childbirth. There is no clear definition of FoB, but it is usually categorised into either primary when occurring before birth or secondary when arising after experiences of traumatic or distressing childbirth [1].

DS can be a consequence of long-term FoB [2], and can also be a risk factor for severe FoB during pregnancy and after childbirth [3–6]. Some women develop a phobic fear, leading to total avoidance of birth because of DS [1, 3, 4]. Several systematic reviews [7–12] and longitudinal studies [10, 13, 14] have reported the prevalence rates of FoB and DS before or after childbirth. Few longitudinal studies have followed the same women to assess these conditions before and after birth.

Rates of FoB and DS vary between countries, with global prevalence rates of 5–30% for antenatal FoB (A-FoB) [1, 7, 12–16] and 5–34% for antenatal DS (A-DS) [17–20], whereas postnatal FoB (P-FoB) ranges between 5 and 20% [16, 21, 22] and postnatal DS (P-DS) between 4 and 25% [23]. Most studies have reported lower FoB and DS rates during the postpartum period than during pregnancy [10, 11, 16, 22, 24].

FoB can be associated with expectations and experiences of labour such as pain or operative procedures; fear of being incapable of giving birth; perceived less childbirth knowledge; not having a voice in decision making; the outcome of labour, including the woman’s and baby’s wellbeing; previous obstetric complications; expectations of parenthood or fear of becoming a parent, low social support and experiences of a non-supportive partner and/or health care providers [22, 25–30]. Severe FoB can affect childbirth, for instance, leading to increased labour duration or a more significant number of obstetric interventions and inducing fear of subsequent pregnancy [25, 28, 31, 32]. DS is more complicated since personality resources can have an impact together with life events. Several factors may be related to DS, such as being single, divorced or separated, not having a formal education, deployment, young age, having a large family, financial instability, previous caesarean section, history of miscarriage, unplanned pregnancy, lack of preparation for childbirth, being a victim of gender-based violence, lack of social support, lack of parenting knowledge and low self-efficacy [18, 19, 33–36]. DS can interrupt women’s daily activities, mother-infant bonding and infant sleeping patterns [24, 37, 38].

Studies have explored the prevalence rates of common perinatal mental disorders (CPMDs) in Tanzania, including DS, anxiety and post-traumatic stress disorders [19, 39–43]. However, the rates differ between regions. FoB after childbirth is understudied. Some studies have indicated that FoB and DS can be present in the same woman [5, 44]. A study in Tanzania revealed an association between FoB and DS during pregnancy and investigated associations with sociodemographic and obstetric factors [40]. Screening of mental health disorders is limited during routine prenatal care. One study in Dar es Salaam, Tanzania, indicated that in women with the possibility of having mental health disorders, their symptoms were not identified and diagnosed if they didn’t raise their concerns by themselves. Probably due to inadequate knowledge and skills among healthcare providers in identifying and managing prenatal mental health problems [43]. We could not identify an informational package which is integrated into routine prenatal care guidelines to guide healthcare providers in providing women with information about CPMDs. However, one cluster-randomised controlled trial in 12 villages in rural Ifakara, Tanzania, was done to assess the effect of a community health worker-delivered intervention on maternal depressive symptoms in rural Tanzania [45]. To our knowledge, no study has compared the prevalence rates of FoB and DS before and after childbirth in the same cohort of women and any associations with other factors. Hence, this study aimed to assess the prevalence rates of FoB and DS after birth, including patterns of FoB and DS from pregnancy to postpartum. We also assessed predictors for the development and persistence of FoB and DS in women after childbirth.

## Methods and materials

### Study design and setting

This was a longitudinal study among a cohort of pregnant women followed up after childbirth. We assessed patterns among women who recovered from A-FoB and/or A-DS and did not experience P-FoB and/or P-DS, women who did not experience A-FoB and/or A-DS but experienced P-FoB and/or P-DS, and women who had FoB and/or DS that persisted from pregnancy throughout the postpartum period.

We carried out this study in six government-owned health facilities in the Kisarawe and Mkuranga districts in the Pwani region, Tanzania. The Pwani region is located in the Eastern zone and encompasses seven districts: Kisarawe, Mkuranga, Mafia, Bagamoyo, Kibiti, Rufiji and Kibaha [46]. The Kisarawe district has 40 health facilities, one district hospital, three governmental health centres and 36 dispensaries. Among 36 dispensaries, 32 are government-owned, and four are private [47]. The Mkuranga district has 57 health facilities: one hospital, six health centres (two governmental and four privately owned) and 50 dispensaries (37 governmental and 13 privately owned). We randomly chose two health centres and one district hospital from each district. Data collection took place at the selected health facilities’ reproductive and child health clinics. Under Tanzanian policy, maternal and child health services at public health facilities are offered free of charge. During the data collection period, district hospitals had vaginal and caesarean birth facilities, while health centres provided services only to women giving birth vaginally. In case of childbirth complications, women were referred to higher-level health facilities for advanced management.

### Study participants and procedure

**Fig 1:**
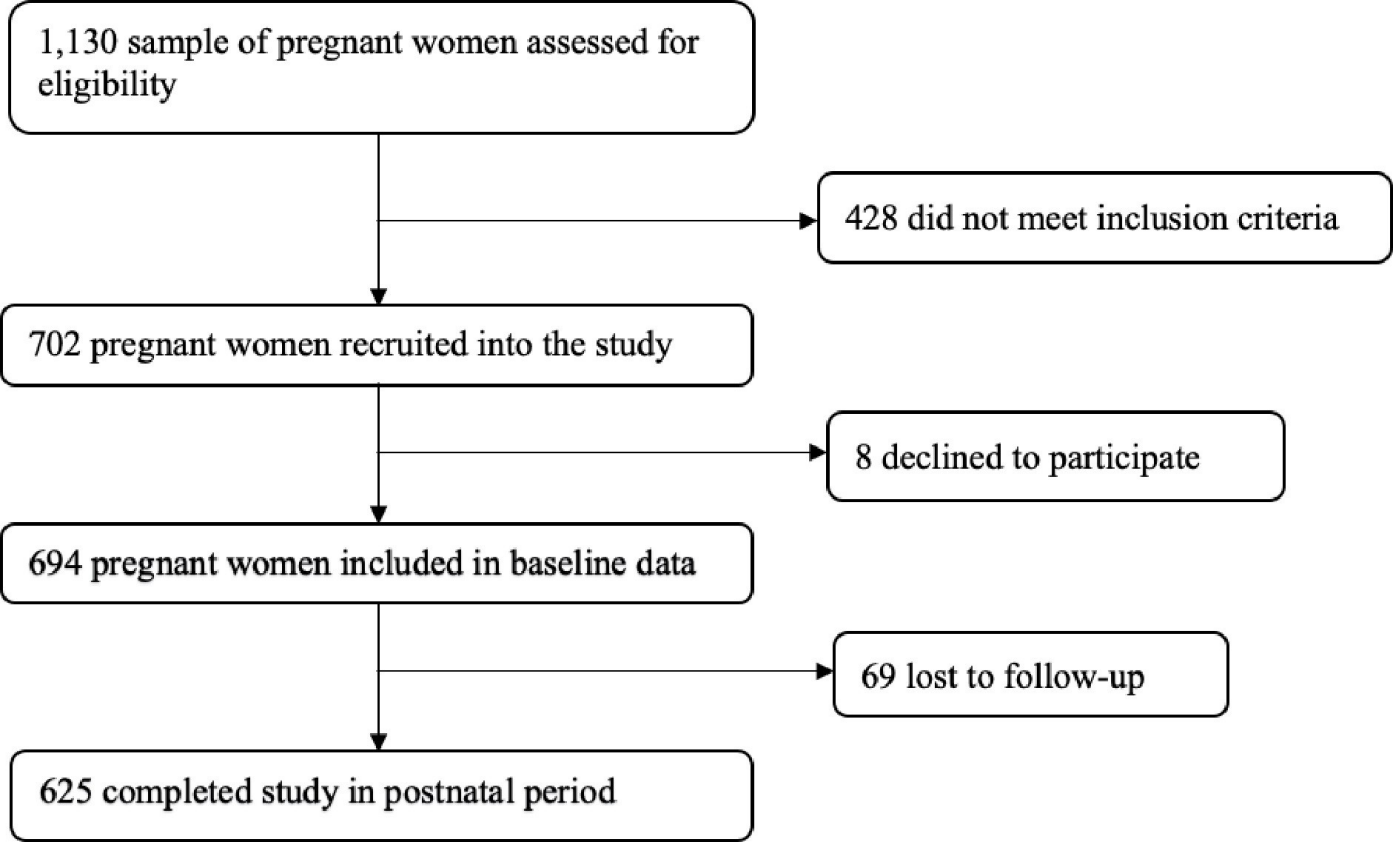
Flowchart for recruitment and follow-up of participants.

We consecutively recruited and interviewed 694 pregnant women who came for routine antenatal care at the selected health facilities from September 2018 to July 2019. We computed a sample size using the two-proportion formula in Epi Info 7 StatCal, with a power of 80% and a significance level of 0.05 with two tails. A minimum sample of 616 pregnant women would need to be enrolled in this study. Inclusion criteria were: resident of the chosen study setting; attending the same health facility for antenatal and postnatal care; pregnancy of at least 32 weeks of gestation; no previous caesarean section; anticipating vaginal birth; Kiswahili speaker. After childbirth, women were followed up and interviewed between 4–6 weeks postnatally at reproductive and child health clinics, using the same approach and tools as during antenatal data collection. However, some women did not return to the same health facility after childbirth, which meant that postnatal data were collected at health facilities and within the community. Recruitment, interviews and follow-ups took place concurrently, and the final sample of women who completed participation numbered 625 (Fig 1).

We trained six nurse-midwives, who were not formally hired during data collection, as research assistants to collect data. An interviewer-administered questionnaire in the Kiswahili language was the primary data collection approach. We developed a visual scale to enable participants to rate their expectations and experiences in childbirth using the Wijma Delivery and Expectation Questionnaire versions A &B (W-DEQ-A&B). This would decrease the risk of having differing verbal explanations for response items from different research assistants.

### Instruments and measures

#### Sociodemographic and obstetric characteristics

We asked participants questions regarding sociodemographic (age, education level, occupation, income and marital status) and obstetric characteristics (gravidity, parity, pregnancy status and complications in previous pregnancies). Obstetric questions during pregnancy referred to the history of the most recent previous pregnancy, and those after childbirth referred to index pregnancy/childbirth history.

#### Wijma delivery expectancy/experience questionnaire versions A and B

We used W-DEQ-A and W-DEQ-B [48] to assess participants’ A-FoB and P-FoB, respectively. The questionnaire was developed by Wijma in 1998 in Swedish to assess women’s cognitive appraisal of the childbirth process during pregnancy (version A) and after childbirth (version B) [48]. The original versions had 33 items measured on a 6-point Likert scale ranging from 0 (not at all) to 5 (extremely) and was hypothesised to be a unidimensional tool [48]. The minimum score is 0, and the maximum is 165. The higher the total score, the greater the FoB. The tool developer proposed cut-off points as follows: a score of ≤ 37 reflects a low level of fear, a score of 38–65 reflects a moderate level of fear, a score of 66–84 reflects a high level of fear, and a score of ≥ 85 indicates a severe level of fear [49]. We defined high FoB as ≥ 66 and low FoB as < 66 [49]. In this study, FoB was considered present in those high-scoring FoBs and no FoB in those low-scoring FoBs.

The original tools have been reported to have good reliability and validity [48]. The internal consistency of the W-DEQ-A and W-DEQ-B was found with Cronbach’s alpha coefficients of 0.93 and 0.94, respectively. Both English versions of W-DEQ were translated into Kiswahili, piloted and underwent a validation process in Tanzania (Massae, Larsson, Leshabari, Mbekenga, Pembe and Svanberg. Fear of childbirth: validation of the Kiswahili version of Wijma Delivery Expectancy/Experience questionnaire versions A and B in Tanzania. Forthcoming). Internal consistency reliability of the translated W-DEQ-A and W-DEQ-B was 0.83 and 0.85, respectively.

#### Edinburgh postnatal depression scale

The Edinburgh Postnatal Depression Scale (EPDS) [50] was used to assess DS during pregnancy or/and after childbirth. Cox and colleagues developed the tool in 1986 [50]. The EPDS has ten items on a 4-point Likert scale ranging from 0 to 3. The lowest score is 0, and the maximum is 30. The higher the score, the higher the risk of having DS. The cut-off scores from the original tools are: ≥ 10, presence of DS; ≥ 13, depressive illness of increasing severity [50]. In this study, scoring ≥ 10 was considered to indicate DS and < 10 to indicate no DS [41, 51, 52]. During development, the tool has shown to be valid and reliable, with a Cronbach’s alpha coefficient of 0.88. The Kiswahili version of EPDS was adapted from one validated in Kenya with an acceptable Cronbach’s alpha of 0.78 [20]. Our study had a Cronbach’s alpha of 0.85 at both time points.

## Ethical approval and consent to participate

We obtained ethical clearance from the Muhimbili University of Health and Allied Sciences, Senate Research and Publication Committee, with reference number 2018-03-09/AEC/Vol.XII/91. Permission to collect data in the selected health facilities was granted by the Regional Administrative Secretary in the Pwani region, Tanzania. Before the inclusion of women in this study, they were informed that their participation was voluntary and that they were allowed to withdraw at any time without being asked about their reasons. Qualified participants provided written informed consent before their participation. Participants under 18 years of age were considered emancipated minors and allowed to sign the consent form without their parents/guardians [53]. Confidentiality and anonymity were ensured throughout data collection. Participants under 18 years were considered emancipated minors and allowed to sign the consent form without their parents/guardians [53]. Confidentiality and anonymity were ensured throughout data collection.

### Data analysis

We analysed data using the Statistical Package for the Social Sciences, version 26. The outcome variables were continuous. We dichotomised the total scores of the outcome variables into two groups, participants with P-FoB Vs those with no P-FoB and participants with P-DS Vs those with no P-DS, for further analysis. Descriptive statistics were used to describe the proportions of sociodemographic and obstetric variables and the prevalence rates of FoB and DS before and after childbirth. The proportion difference analysis between A-FoB and P-FoB was performed using a paired proportion McNemar test. Binary logistic regression was used for both univariable and multivariable analyses. During analysis, the groups were treated as both independent and dependent. Univariable analysis with odds ratios (ORs) and 95% confidence intervals (CIs) of ORs were used to explore and measure P-FoB and P-DS predictors. All variables with a significant association (univariable logistic regression) of p < 0.2 with P-FoB and P-DS were entered into the multivariable logistic regression models using an ENTER approach: independent variables were entered into each model, one after another. For the final models, p < 0.05 was considered statistically significant. Adjusted odds ratios (aORs) and 95% CIs were used. The outcomes of interest were grouped as (1) FoB after childbirth vs no FoB after birth, DS after childbirth vs no DS after childbirth, (2) No FoB or DS during pregnancy or after childbirth vs FoB after childbirth only and persisting FoB or DS after childbirth only and persisting DS.

## Results

### Description of the population

Of 694 eligible pregnant women who agreed to participate, 625 were followed up in a second interview (Fig 1). In this paper, we have included the participants who provided information during pregnancy and at 4–6 weeks after childbirth to compare findings before and after and for longitudinal analysis. Sixty-nine participants (9.9%) were lost to follow-up due to poor communication (some women did not own mobile phones and others were unreachable during the follow-up), travel after birth or geographical problems in reaching them. Among those lost to follow-up, eleven (15.9%) withdrew from the study due to various reasons like infant death or lack of permission from their male partners to continue with the study. Three questionnaires for measuring FoB after childbirth were missing three responses each on how women felt when labour became most intense. The missing items were from women who underwent caesarean sections. To address this, the imputation of data was performed using the mean substitution technique.

The age of women who completed the study ranged between 14 and 46 years, with a median of 26 years and an interquartile range of 24 years. The majority (72.0%) were married and had primary education (61.4%). More than 50% of participants were multiparous, and 52.3% did not experience any obstetric complications during the index pregnancy or childbirth. More than 80% of women gave birth at health facilities, and most babies were full-term and healthy. The majority of the women (52.3%) were socially supported by their male partners. See Table 1 for further details.

**Table 1:** Sociodemographic and obstetric characteristics of the participants.

Variables	Total cohort	Postnatal follow-up	Lost to follow-up	[Difference between those followed up (625) and those lost to follow-up (69)]
	n (%)	(n%)	n (%)	P-value
**Index pregnancy and birth variables**	**(n = 694)**	**(n = 625)**	**(n = 69)**	
**Age groups**				
≤ 20 years	139 (20.0)	116 (18.6)	17 (24.6)	0.127
21–30 years	358 (51.6)	323 (51.7)	37 (53.6)	
≥ 31 years	197 (28.4)	186 (29.8)	15 (21.7)	
**Education level**				
No formal education	137 (19.7)	124 (19.4)	13 (18.8)	0.628
Primary education	429 (61.8)	384 (61.4)	45 (65.2)	
Secondary education or higher	128 (18.4)	117 (18.7)	11 (15.9)	
**Occupation**				
Employed	490 (70.6)	445 (71.2)	45 (65.2)	0.330
Not employed/not earning	204 (29.4)	180 (28.8)	24 (34.8)	
**Marital status**				
Married	508 (73.2)	450 (72.0)	58 (84.1)	**0.044**
Not married	186 (26.8)	175 (28.0)	11 (15.9)	
* Income adequacy				
Adequate	304 (43.8)	268 (42.9)	36 (52.2)	0.160
Inadequate	390 (56.2)	357 (57.1)	33 (47.8)	
**Pregnancy status**				
Planned	471 (67.9)	424 (67.8)	47 (68.1)	0.963
Unplanned	223 (32.1)	201 (32.2)	22 (31.9)	
**Parity**				
Nullipara	190 (27.4)	0 (0.0)	19 (27.5)	
Primipara	157 (22.6)	172 (27.5)	17 (24.6)	0.902
Multipara	347 (50.0)	453 (72.5)	33 (47.8)	
**Preferred sex of the baby**				
Female	244 (35.2)	218 (34.9)	26 (37.7)	
Male	204 (29.4)	190 (30.4)	14 (20.3)	0.198
Any sex	246 (35.4)	217 (34.7)	29 (42.0)	
**Previous pregnancy/birth and index pregnancy/birth variables**	**(n = 504)**	**(n = 625)**	**(n = 50)**	
** Ever having experienced obstetric complications in a previous pregnancy/birth or index pregnancy/birth				
Yes	141 (28.0)	298 (47.7)	14 (28.0)	0.509
No	363 (72.0)	327 (52.3)	36 (72.0)	
** Mode of delivery in previous birth/index baby				
Vaginal birth without episiotomy	498 (98.8)	550 (88.0)	46 (92.0)	
Vaginal delivery with episiotomy	4 (0.8)	0 (0.0)	2 (4.0)	
Instrument-aided delivery	2 (0.4)	0 (0.0)	2 (4.0)	
Caesarean section	0 (0.0)	75 (12.0)	0 (0.0)	***
** place of birth in most recent previous birth/index birth				
Home/birth before arrival	54 (10.7)	25 (4.0)	3 (6.0)	
Dispensary	22 (4.4)	10 (1.6)	3 (6.0)	
Health centre	144 (28.6)	217 (34.7)	14 (28.0)	0.881
Hospital	284 (56.3)	373 (59.7)	30 (60.0)	
** Social support in previous/index birth				
**Male partner/husband**				
Yes	312 (61.9)	327 (52.3)	28 (56.0)	0.256
No	192 (38.1)	298 (47.7)	22 (44.0)	
**Parents**				
Yes	261 (51.8)	316 (50.6)	29 (58.0)	0.348
No	243 (48.2)	309 (49.4)	21 (42.0)	

Percentages were calculated based on the number of respondents for each variable and category/timeline. First-time pregnant women were excluded from the analysis of questions involving past pregnancy experiences.

* Based on participant responses, i.e., self-perceived income adequacy.

** information provided during the antenatal period referred to the most recent previous pregnancy/birth. Information provided after childbirth referred to the index pregnancy/index delivery.

*** P-value not computed because some cells had an expected count of less than five, and some variables were constant.

### Information on index birth and baby

Table 2 indicates that a large proportion (81.8%) of women had less than 12 hours pass between admission and birth. Participants who were lost to follow-up and hence excluded from the longitudinal and comparative analysis were younger, more often employed, married, and socially supported by male partners than those included. Also, participants who were lost to follow-up were less likely to have A-FoB and A-DS.

**Table 2:** Information of the index baby.

Variables	Postnatal follow-up (n = 625)	
	n	%
**Sex of index baby**		
Female	308	49.3
Male	317	50.7
* Completed weeks of gestation		
Preterm (< 37 weeks)	93	14.9
Full-term (38–42 weeks)	512	81.9
Post-term (> 42 weeks)	20	3.2
* Birthweight		
Low birthweight	24	3.8
Normal birthweight	490	78.4
High birthweight	111	17.8
**Status of index baby immediately after birth**		
Healthy	580	92.8
Ill	33	5.3
Stillbirth	7	1.1
Early neonatal death	5	0.8
** Time between admission and birth		
≤ 12 hours	511	81.8
> 12 hours	114	18.2

Percentages were calculated based on the number of respondents for each variable and category/timeline.

* Information from the participants and antenatal and under-five cards.

** Based on participant responses, i.e., hours spent in the labour ward.

The overall scores for P-FoB varied between 0 and 96, with a median score of 47.0. The prevalence rates of A-FoB and P-FoB were 16.0% and 13.9%, respectively, and there was an insignificant decrease of P-FoB by 2.1%. A total of 40 participants (6.4%) had persistent fear during pregnancy and postpartum. On the other hand, 60 participants (9.6%) had fear during pregnancy which resolved after childbirth, while 47 (7.5%) had no fear during pregnancy but became fearful after birth (Table 3).

**Table 3:** Patterns of fear of childbirth and depressive symptoms during pregnancy and after childbirth.

		**P-FoB**			
		**No fear**	**Fear**	**Total**	**P-value**
**A-FoB**	**No fear**	478 (76.5)	47 (7.5)	525 (84.0)	
n (%)	**Fear**	60 (9.6)	40 (6.4)	100 (16.0)	
**Total**		538 (86.1)	87 (13.9)	**625 (100.0)**	0.246
		**P-DS**			
		**No depressive symptoms**	**Depressive symptoms**	**Total**	**P-value**
**A-DS**	**No depressive symptoms**	485 (77.6)	26 (4.2)	511 (81.8)	
n(%)	**Depressive symptoms**	87 (13.9)	27 (4.3)	114 (18.2)	
**Total**		572 (91.5)	53 (8.5)	**625 (100.0)**	**< 0.001**
		**P-DS**			
		**No depressive symptoms**	**Depressive symptoms**	**Total**	**P-value**
**P-FoB**	**No fear**	503 (80.5)	35 (5.6)	538 (86.1)	
n(%)	**Fear**	69 (11.0)	18 (2.9)	87 (13.9)	
**Total**		572 (91.5)	53 (8.5)	**625 (100.0)**	**< 0.001**

A-DS = Antenatal depressive symptoms; A-FoB = Antenatal fear of childbirth; P-DS = postnatal depressive symptoms; P-FoB = Postnatal FoB

The overall scores for P-DS were 0–23. The median (IQR) for P-DS was 1 (4). The prevalence rate of A-DS was 18.2%, and that of P-DS was 8.5%. The proportion decreased significantly after childbirth (p < 0.001). Twenty-seven women (4.3%) had DS both during pregnancy and after childbirth. A total of 87 women (13.9%) had DS during pregnancy which resolved after birth, whereas 26 (4.2%) had no DS during pregnancy but developed DS after childbirth (Table 3).

### Predictors of P-FoB and P-DS

Tables 4 and 5 display different logistic regression models for univariable and multivariable analyses on P-FoB and P-DS as outcomes when the groups were treated as independent and dependent, respectively. The variables associated with FoB and DS were entered into the multivariable analysis models, one after another.

**Table 4:** Relationships between fear after childbirth and sociodemographic and obstetric factors.

	Fear of childbirth					
	Fear after childbirth		Predictors for developing fear postnatally		Predictors for persisting fear of childbirth	
	n = 87/625 (13.9%)		n = 47/525 (9.0%)		n = 40/518 (7.7%)	
	Unadjusted	Adjusted	Unadjusted	Adjusted	Unadjusted	Adjusted
Variables	n (%) OR (95% CI)	OR (95% CI)	n (%) OR (95% CI)	OR (95% CI)	n (%) OR (95% CI)	OR (95% CI)
**Occupation**						
Employed	51 (58.6) Ref	Ref	30 (63.8) Ref		21 (52.5) Ref	Ref
Not employed	36 (41.4) 1.93 (1.21–3.08)*	1.69 (0.99–2.88)	17 (36.2) 1.53 (0.82–2.87)		19 (47.5) 2.45 (1.27–4.70)*	2.31 (1.16–4.59)*
**Parity**						
Primipara	33 (37.9) Ref		31 (66.0) Ref		13 (32.5) Ref	
Multipara	54 (62.1) 0.57 (0.36–0.92)*	1.03 (0.53–1.99)	16 (34.0) 0.46 (0.25–0.86)*		27 (67.5) 0.70 (0.35–1.41)	
**Ever having experienced obstetric complications.**						
Yes	33 (37.9) Ref	Ref	14 (29.8) Ref	Ref	23 (57.5) Ref	Ref
No	54 (62.1) 0.53 (0.31–0.93)*	0.44 (0.23–0.83)*	33 (70.2) 0.35(0.18–0.67)*	0.37 (0.19–0.72)*	17 (42.5) 0.41 (0.21–0.79)*	0.45 (0.22–0.88)*
**Planned pregnancy**							
No	30 (34.5) Ref	Ref	15 (31.9) Ref		21 (52.5) Ref	Ref
Yes	57 (65.5) 0.48 (0.30–0.77)*	0.71 (0.41–1.21)	32 (68.1) 0.64 (0.33–1.22)		19 (47.5) 0.27 (0.14–0.52)**	0.28(0.14–0.55)**
**Mode of delivery for index baby**						
Vaginal delivery	70 (80.5) Ref	Ref	36 (76.6) Ref	Ref	34 (85.0) Ref	
Caesarean section	17 (19.5) 2.01 (1.11–3.65)*	2.04 (1.02–4.09)*	11 (23.4) 2.39 (1.15–4.99)*	1.94 (0.88–4.24)	6 (15.0) 1.39 (0.56–3.45)	
**Time between admission and delivery**						
≤ 12 hours	63 (72.4) Ref	Ref	31 (66.0) Ref	Ref	32 (80.0) Ref	
> 12 hours	24 (27.6) 1.89 (1.13–3.19)*	1.69 (0.94–3.09)	16 (34.0) 2.61 (1.36–4.99)*	2.07 (1.03–4.15)*	8 (20.0) 1.26 (0.56–2.84)	
**Fear of childbirth during pregnancy**						
No	47 (54.0) Ref	Ref				
Yes	40 (46.0) 6.78(4.11–18.11)**	5.86 (3.37–10.21)**	NA	NA	NA	NA
**Depressive symptoms during pregnancy**						
No	58 (66.7) Ref	Ref	36 (76.6) Ref		22 (55.0) Ref	
Yes	29 (33.3) 2.67 (1.61–4.40)**	1.43 (0.79–2.61)	11 (23.4) 1.81 (0.88–3.73)		18 (45.0) 4.85 (2.47–9.51)**	
**Depressive symptoms after childbirth**						
No	69 (79.3) Ref	Ref	38 (80.9) Ref	Ref	31 (77.5) Ref	Ref
Yes	18 (20.7) 3.75 (2.01–6.98)**	3.13 (1.51–6.48)*	9 (19.1) 3.42 (1.52–7.69*	3.48 (1.50–8.05)*	9 (22.5) 4.19 (1.83–9.57)**	3.73 (1.55–8.98)*

*p < 0.05,

**p < 0.001; NA = Not applicable to this group;

*** Self-reported information

Gaps in some variables indicate that the P-values in the univariable analysis were not < 0.05. Therefore, these variables were not entered into the multivariable models for further analysis

**Table 5:** Relationships between depressive symptoms after childbirth and sociodemographic and obstetric factors.

	Depressive symptoms					
	Depressive symptoms after childbirth		Predictors of depressive symptoms postnatally		Predictors of persisting depressive symptoms	
	n = 53/625 (8.5%)		n = 26/511 (5.1%)		n = 27/512 (5.3%)	
	Unadjusted	Adjusted	Unadjusted	Adjusted	Unadjusted	Adjusted
Variables	n (%) OR (95% CI)	OR (95% CI)	n (%) OR (95% CI)	OR (95% CI)	n (%) OR (95% CI)	OR (95% CI)
***Income adequacy						
Adequate	17 (32.1) Ref		10 (38.5) Ref		7 (25.9) Ref	
Inadequate	36 (67.9) 1.66 (0.91–3.02)		16 (61.5) 1.39 (0.62–3.11)		20 (74.1) 2.47 (1.03–5.96)*	
**Marital status**						
Married	34 (64.2). Ref		17 (65.4) Ref		14(51.9) Ref	Ref
Single	19 (35.8) 1.49 (0.83–2.69)		9 (34.6) 0.88 (0.35–2.25)		13 (48.1) 2.73 (1.25–5.97)*	2.59 (1.02–6.59)*
**Parity**						
Primipara	9 (17.0) Ref		7 (26.9) Ref		2 (7.4) Ref	
Multipara	44 (83.0) 1.95 (0.93–4.08)		19 (73.1) 1.08 (0.44–2.63)		25 (92.6) 4.97 (1.16–21.27)*	
**Planned pregnancy**						
No	12 (22.6) Ref	Ref	9 (34.6) Ref		13 (48.1) Ref	
Yes	41 (77.4) 0.51 (0.28–0.90)*	1.44 (0.71–2.90)	17 (65.4) 0.62 (0.27–1.43)		14 (51.9) 0.35 (0.16–0.77)*	
**Ever having experienced obstetric complications.**						
Yes	16 (30.2) Ref	Ref	9 (34.6) Ref		9 (33.3) Ref	
No	37 (69.8) 0.40 (0.22–0.76)*	0.50 (0.25–1.01)	17 (65.4) 0.61 (0.21–1.73)		18 (66.7) 0.34 (0.15–0.78)**	
**Social support from parents**						
No	35 (66.0) Ref	Ref	15 (57.7) Ref		20 (74.1) Ref	**Ref**
Yes	18 (34.0) 0.47 (0.26–0.85)*	0.43 (0.23–0.82)*	11 (42.3) 0.58 (0.24–1.41)		7 (25.9) 0.33 (0.14–0.79)*	0.28 (0.11–0.69)*
**Status of index baby immediately after birth**						
Healthy	41 (77.4) Ref	Ref	20 (76.9) Ref	Ref	21 (77.8) Ref	
Ill/stillbirth/early neonatal death	12 (22.6) 4.78 (2.29–9.95)**	4.23 (1.88–9.79)**	6 (23.1) 5.29 (1.96–14.31)*	4.87 (1.76–13.47)*	6 (22.2) 5.04 (1.88–13.57)*	
**Depressives symptoms during pregnancy**						
No	26 (49.1) Ref	Ref				
Yes	27 (50.9) 5.79 (3.23–10.39)**	4.81 (2.59–8.93)**	NA	NA	NA	NA
**Fear of childbirth during pregnancy**						
No	40 (75.5) Ref		21 (80.8) Ref		19 (70.4) Ref	
Yes	13 (24.5) 1.81 (0.93–3.53)		5 (19.2) 1.66 (0.60–4.55)		8 (29.6) 2.93 (1.23–6.98)*	
**Fear after childbirth**						
No	33 (66.0) Ref	Ref	16 (61.5) Ref	Ref	17 (63.0) Ref	
Yes	17 (34.0) 3.75 (2.01–6.98)**	2.79 (1.40–5.54)*	10 (38.5)4.60(1.99–10.62)**	3.59 (1.46–8.87)*	10 (37.0) 5.12 (2.22–11.79)**	

*p < 0.05,

**p < 0.001; NA = Not applicable to this group;

*** Self-reported information

Gaps in some variables indicate that the P-values in the univariable analysis were not < 0.05. Therefore, these variables were not entered into the multivariable models for further analysis.

Several predictors were significantly associated with P-FoB. Having A-FoB, P-DS were the strongest predictors of P-FoB. Other predictors were giving birth by caesarean section and having experienced obstetric complications in the index pregnancy. Detailed findings are provided in Table 4.

### Predictors for developing FoB and DS

The odds of developing FoB only after childbirth compared with no FoB at either timepoint were higher in women who had experienced obstetric complications in a previous pregnancy, had more than twelve hours pass between admission and childbirth or had P-DS. The significant predictors of having both A-FoB and P-FoB compared with no FoB at either timepoint were being unemployed, having an unplanned pregnancy, ever having experienced any obstetric complication and having P-DS. More results are shown in Table 4.

### Supplemental analysis of the 69 participants lost to follow-up

A supplemental analysis was performed of the 69 participants lost to follow-up to evaluate if this loss would affect the results. Most of those lost to follow-up were younger and more often employed and married. Also, the majority had planned their pregnancy, were multipara and were socially supported by a male partner. Being socially supported by a male partner in previous childbirth was associated with less fear during pregnancy. Women with secondary education were more likely to have DS than those less educated. The rest of the variables were not associated with FoB or DS. Details of FoB and DS predictors during pregnancy are found elsewhere [40].

As shown in Table 5, several predictors were associated with P-DS. The predictors were: lack of parental social support after delivery; having given birth to an ill or dead baby; having A-DS or P-FoB. P-FoB and giving birth to an ill baby were predictors of P-DS only compared with no DS at either timepoint.

Predictors associated with having both A-DS and P-DS compared with no DS at either timepoint were: being single and not having social support from parents.

## Discussion

This study described patterns of FoB and DS among women who were followed up during their postpartum period in Tanzania. Findings showed that some women experienced FoB (6.4%) and DS (4.3) during pregnancy and after childbirth, while some did not experience it while pregnant but developed after childbirth. The odds of women developing P-FoB were higher among women with more than twelve hours passed between admission and childbirth. The odds of developing P-DS were higher for women with P-FoB while being single and having experienced a lack of parent social support in previous childbirth were associated with increased odds of persisting DS. Having experienced obstetric complications in previous childbirth and P-DS were cross-cutting predictors in women for developing FoB and having P-FoB or persisting FoB. Giving birth to an ill infant predicted developing DS and having P-DS.

The current study determined that P-FoB and P-DS prevalence rates among Tanzanian women did not differ from those in other parts of the world. Compared with other studies in Tanzania, A-DS prevalence rates were lower than those reported in Mwanza [39], while P-DS rates were almost the same as those reported from Kilimanjaro [38]. The FoB variation was within global ranges, despite global differences in income level, cultural context, birth rates, health systems and other factors [16, 17, 21, 22]. Childbirth is a natural process accompanied by experiences such as labour pain [54, 55], loss of body control [56] and other unforeseeable events, which could explain why women from different backgrounds and nationalities can share similar rates of FoB and DS.

The decrease of FoB and DS over time from pregnancy to postpartum is significant when dealing with women seeking antenatal or postnatal care. The decline may follow a natural pathway, as some women may have many emotional disturbances and expectations before birth, including fear of the unknown. Generally, previous studies have indicated that FoB [16, 22, 57] and DS [11, 24, 58] are more prevalent during pregnancy than postpartum. However, a study by Fenwick and colleagues in Australia [59] reported that women delivered through emergency caesarean sections had higher levels of P-FoB than A-FoB. This could support our findings since most women in this study had a vaginal birth. Despite the decrease, FoB did not resolve entirely in some women.

Similarly, studies in other parts have shown that women with A-FoB and A-DS are likely to have FoB [22, 60] and DS [17, 61] after childbirth. This suggests that pregnancy monitoring and addressing the most severe emotional, psychological and physical issues, which could aggravate FoB and DS at both timepoints, are essential. We found that not all women with A-DS and A-FoB presented with DS and FoB after childbirth. On the other hand, some women developed these conditions only after childbirth. This indicates that DS and FoB can occur at any time from birth to the postpartum period. These findings might interest healthcare providers, particularly midwives, in raising awareness that FoB and DS can change over time. Thorough assessment and care throughout pregnancy and the postpartum period are crucial for alleviating potential adverse childbirth outcomes. This was in line with a systematic review report that revealed that DS could develop throughout the antepartum and postpartum periods [8].

The finding that FoB is associated with DS reminds healthcare providers that these conditions can coexist. Having P-DS increased the risks of P-FoB and the other way around. Thus, a thorough assessment of both conditions during pregnancy and after childbirth is paramount for early identification and timely intervention. The findings were similar to those in previous longitudinal studies, which suggested that FoB is among the strongest predictors of P-DS [2, 62, 63]. Contrary to a study from Iran assessing anxiety and FoB as predictors of postnatal depression in nulliparous women, A-FoB was not a significant predictor of P-DS [64]. The difference might be explained by the timing of assessing prenatal and postpartum FoB and DS and/or contextual factors.

Our study revealed a history of obstetric complications in the most recent previous pregnancy and childbirth as a common cross-cutting predictor for developing P-FoB and having FoB at both timepoints. Childbirth complications can be unpredictable and vary from one woman to another. Similarly, childbirth expectations during pregnancy differ and can lead to negative childbirth experiences if they are not met. Negative experiences at health facilities can lead to frustration, confusion, anger and lack of physical and emotional control, affecting women’s decisions on future childbirth [22, 65–67]. If a woman’s actual childbirth experience does not match her expectations, it can lead to the development of FoB [31, 68, 69]. Health providers have to be aware of women’s wishes and work to meet their expectations. Timely interventions such as psychological support to prevent complications during pregnancy, childbirth and postpartum will promote positive childbirth experiences.

More than twelve hours pass between admission and childbirth at health facilities could contribute to developing P-FoB. This might be due to an overly long labour time, long waiting and thinking about childbirth outcomes. The findings align with an Indian study showing that women who spent six or more hours at a health facility before childbirth more often had P-FoB [21]. Other studies have found that FoB was a predictor of prolonged labour [31, 59]. This indicates that FoB and duration of labour predict each other. Therefore, it is essential for health care providers to ensure FoB and duration of labour are considered when providing care to women during labour and childbirth.

An ill newborn, a stillbirth or an early neonatal death contributed to developing P-DS, probably because most pregnant women expect healthy babies. This highlights the importance of healthcare providers offering care that meets women’s and their families childbirth expectations. Moreover, our study findings showed that persisting DS appeared to be associated with being single and lacking social support from parents in a previous childbirth. That being a single mother was a predictor of both A-DS and P-DS is not surprising and could be due to a lack of social, financial and psychological support from a male partner. Based on the Tanzanian cultural context, single parenting can also be misinterpreted as an indicator of low status by the surrounding community, accompanied by stigma. However, there is limited literature to support or contradict our findings. Few studies have followed the same cohort of women from pregnancy through birth to postpartum to assess patterns and predictors of developing DS or having persisting DS. We have found studies that determine predictors of A-DS and P-DS independently in various groups of women. For instance, a systematic review [70] and a prospective cohort study [35] reported that single mothers had an increased likelihood of A-DS.

## Clinical implications of the findings

Several factors contribute to FoB and DS before and after childbirth in Tanzania–and possibly other parts of Africa. This reflects the multiple challenges and approaches to meeting women’s expectations and promoting positive childbirth experiences. The findings have provided insight into the magnitude of the problem, influencing factors and the importance of improving the care provided to Tanzanian women. During the antenatal period, FoB and DS screening is paramount in identifying and supporting women at risk of developing mental illnesses and those who have experienced complications during previous pregnancies and childbirth.

The findings also highlight the importance of integrating mental health components into routine antenatal and postnatal care.

## Strengths and limitations

To our knowledge, this is the first study to assess FoB and its relationship to DS in Tanzania. A follow-up study from the third trimester of pregnancy to 6 weeks after childbirth gave us information. It revealed patterns on how different factors can predict FoB and DS at varying timepoints in the same cohort. This could guide the appropriate timing for interventions. Furthermore, the loss to follow-up was small (9.9%). Those omitted from the longitudinal analysis were not significantly different from the original sample recruited during the antenatal period, indicating limited selection bias. We used interviewer-administered questionnaires instead of a self-administered approach which other studies have broadly used to lessen the participants’ struggle to fill in answers by themselves.

Conducting interviews might have biased our findings if the presence of interviewers influenced women’s responses. Furthermore, not including high-risk women during recruitment, such as those who had experienced caesarean childbirth or did not expect vaginal birth, was another limitation of our study. This could underestimate the prevalence rates of FoB and DS. This might also limit the generalisability among women with a previous caesarean section.

## Conclusions

FoB and DS during pregnancy and postpartum were strongly associated with each other. The prevalence rates of FoB and DS were higher among pregnant women than among postnatal mothers. Obstetric complications predicted developing P-FoB and persisting FoB. Having more than 12 hours pass at a health facility before childbirth was associated with higher odds of developing P-FoB; being unemployed was associated with increased odds of having FoB at both timepoints. Given birth to an ill child or experiencing stillbirth was a predictor for developing P-DS while being single and having a lack of social support from parents predicted having DS at both timepoints. Knowing why some women are more likely to develop FoB and DS at different timepoints is essential for timely assessment, early identification and appropriate care to improve women’s health and well-being before and after childbirth. To overcome the prevailing problems, effective interventions need to start during antenatal.

## Supporting information

S1 Table Association between fear after childbirth with sociodemographic and obstetric factors. (DOCX)

S2 Table Association between depressive symptoms after childbirth with sociodemographic and obstetric factors. (DOCX)

## List of abbreviations

A-DS: Antenatal depressive symptoms
A-FoB: Antenatal fear of childbirth
aOR: Adjusted odds ratio
CI: Confidence interval
CPMDs: Common perinatal mental disorders
DS: Depressive symptoms
EPDS: Edinburgh Postpartum Depression Scale
FoB: Fear of childbirth
IQR: Interquartile range
P-FoB: Postpartum fear of childbirth
P-DS: Postpartum depressive symptoms
WEDQ: Wijma Expectancy/Experience Delivery Questionnaire
