# Supplementary material for: Patterns and predictors of fear of childbirth and depressive symptoms over time in a cohort of women in the Pwani region, Tanzania
Source: PLoS One. 2022 Nov 3;17(11):e0277004. doi: 10.1371/journal.pone.0277004 (PMC9632885; doi:10.1371/journal.pone.0277004)
Supplement: S1 Table — (DOCX) [file pone.0277004.s001.docx]

**S1Table. Association between fear after childbirth with sociodemographic and obstetric factors**

| **Factors for developing fear after birth (n=47/525** | | | | | **Factors for persisting fear of childbirth (n=40/518)** | | | |  |
| --- | --- | --- | --- | --- | --- | --- | --- | --- | --- |
|  |  |  |  |  |  |  |  |  |  |
| **Variables** | **Total** | **No** | **Yes** | **P-value** | **Total** | **No** | **Yes** | **P-value** |  |
|  | **n (%)** | **n (%)** | **n (%)** |  | **n (%)** | **n (%)** | **n (%)** |  |  |
| **Occupation** |  |  |  |  |  |  |  |  |  |
| Employed | **NA** |  |  |  | 370 (100) | 349 (94.3) | 21 (5.7) | **0.002** |  |
| Not employed |  |  |  |  | 148 (100) | 129 (87.2) | 19 (12.8) |  |  |
| **Parity** |  |  |  |  |  |  |  |  |  |
| Primipara | 256 (100) | 225 (87.9) | 31(12.1) | **0.015** | **NA** |  |  |  |  |
| Multipara | 269 (100) | 253 (94.1) | 16 (5.9) |  |  |  |  |  |  |
| **Ever experienced obstetric complications** | | | |  |  |  |  |  |  |
| Yes | 248 (100) | 215 (86.7) | 33 (13.3) | **0.001** | 193 (100) | 170 (88.1) | 23 (11.9) | **0.01** |  |
| No | 277 (100) | 263 (94.9) | 14 (5.1) |  | 325 (100( | 308 (94.8) | 17 (5.2) |  |  |
| **Planned pregnancy** | |  |  |  |  |  |  |  |  |
| No | **NA** |  |  |  | 131 (100) | 110 (84.0) | 21 (16.0) | **<0.001** |  |
| Yes |  |  |  |  | 387(100) | 368 (95.1) | 19 (4.9) |  |  |
| **Mode of delivery** | | |  |  |  |  |  |  |  |
| Vaginal delivery | 460 (100) | 424 (92.2) | 36 (7.8) | **0.033** | **NA** |  |  |  |  |
| Caesarean section | 65 (100) | 54 (83.1) | 11 (16.9) |  |  |  |  |  |  |
| **Time between admission and delivery** | | |  |  |  |  |  |  |  |
| ≤ 12 hours | 430 (100) | 399 (92.8) | 31 (7.2) | **0.005** | **NA** |  |  |  |  |
| > 12 hours | 95 (100) | 79 (83.2) | 16 (16.8) |  |  |  |  |  |  |
| **Depressive symptoms during pregnancy** | | |  |  |  |  |  |  |  |
| No | 445 (100) | 409 (91.9) | 36 (8.1) | 0.133 | 431 (100) | 409 (94.9) | 22 (5.1) | **<0.001** |  |
| Yes | 80 (100) | 69 (86.3) | 11 (13.8) |  | 87 (100) | 69 (79.3) | 18 (20.7) |  |  |
| **Depressive symptoms after childbirth** | | |  |  |  |  |  |  |  |
| No | 485 (100) | 447 (92.2) | 38 (7.8) | **0.006** | 478 (100) | 447 (93.5) | 31 (6.5) | **0.002** |  |
| Yes | 40 (100) | 31 (77.5) | 9 (22.5) |  | 40 (100) | 31 (77.5) | 9 (22.5) |  |  |
