# Supplementary material for: Patterns and predictors of fear of childbirth and depressive symptoms over time in a cohort of women in the Pwani region, Tanzania
Source: PLoS One. 2022 Nov 3;17(11):e0277004. doi: 10.1371/journal.pone.0277004 (PMC9632885; doi:10.1371/journal.pone.0277004)
Supplement: S2 Table — (DOCX) [file pone.0277004.s002.docx]

**S2 Table. Association between depressive symptoms after childbirth with sociodemographic**

| **and obstetric factors** |  |  |  |  |  |  |  |  |  |
| --- | --- | --- | --- | --- | --- | --- | --- | --- | --- |
| **Factors for developing depressive symptoms (n=26/511)** | | | | | **Factors for persisting depressive symptoms (n=27/512)** | | | |  |
|  |  |  |  |  |  |  |  |  |  |
| **Variables** | **Total** | **No** | **Yes** | **P-value** | **Total** | **No Yes** | | **P-value** |  |
|  | **n (%)** | **n (%)** | **n (%)** |  | **n (%)** | **n (%)** | **n (%)** |  |  |
| **Marital status** | |  |  |  |  |  |  |  |  |
| Married | **NA** |  |  |  | 376 (100) | 362 (96.3) | 14 (3.7) | **0.013** |  |
| Single |  |  |  |  | 136 (100) | 123 (90.4) | 13 (9.6) |  |  |
| **Parity** |  |  |  |  |  |  |  |  |  |
| Primipara | **NA** |  |  |  | 140 (100) | 138 (98.6) | 2 (1.4) | **0.014** |  |
| Multipara |  |  |  |  | 372 (100) | 347 (93.3) | 25 (6.7) |  |  |
| **Social support from parents** | | |  |  |  |  |  |  |  |
| No | **NA** |  |  |  | 256 (100) | 236 (92.2) | 20 (7.8) | **0.016** |  |
| Yes |  |  |  |  | 256 (100) | 249 (97.3) | 7 (2.7) |  |  |
| **Status of index baby** | |  | |  |  |  |  |  |  |
| Healthy | 479 (100) | 459 (95.8) | 20 (4.2) | **0.004** | 480 (100) | 459 (95.6) | 21 (4.4) | **0.004** |  |
| Ill/stillbirth/early neonatal death | 32 (100) | 26 (81.3) | 6 (18.8) |  | 32 (100) | 26 (81.3) | 6 (18.8) |  |  |
| **Fear after childbirth** | |  | |  |  |  |  |  |  |
| No | 443 (100) | 427 (96.4) | 16 (3.6) | **<0.001** | **NA** |  |  |  |  |
| Yes | 68 (100) | 58 (85.3) | 10 (14.7) |  |  |  |  |  |  |
